# Range Sidelobe Suppression Using Complementary Sets in Distributed Multistatic Radar Networks

**DOI:** 10.3390/s18010035

**Published:** 2017-12-25

**Authors:** Jiahua Zhu, Xuezhi Wang, Yongping Song, Xiaotao Huang, Bill Moran

**Affiliations:** 1 College of Electronic Science, National University of Defense Technology, Changsha 410073, China; sypopqjkl@163.com (Y.S.); xthuang@nudt.edu.cn (X.H.); 2 School of Engineering, RMIT University, Melbourne VIC 3000, Australia; xuezhi.wang@rmit.edu.au (X.W.); wmoran@unimelb.edu.au (B.M.); 3 Department of Electrical and Electronic Engineering, The University of Melbourne, Melbourne VIC 3010, Australia; 4 Collaborative Innovation Center of Information Sensing and Understanding, Changsha 410073, China

**Keywords:** complementary sets, distributed multistatic radar, range sidelobe suppression

## Abstract

We propose an alternative waveform scheme built on mutually-orthogonal complementary sets for a distributed multistatic radar. Our analysis and simulation show a reduced frequency band requirement for signal separation between antennas with centralized signal processing using the same carrier frequency. While the scheme can tolerate fluctuations of carrier frequencies and phases, range sidelobes arise when carrier frequencies between antennas are significantly different.

## 1. Introduction

Distributed multistatic radar, as a special case of multi-input multi-output (MIMO) radar, is widely applied for illumination of targets in a range of scenarios and various types of target tracking. In particular, it is currently used in ultra-wideband (UWB) radar for the detection of human targets [1,2,3], blind selection of representative observations [4], multiperson tracking [5] and indoor tracking [6]. One of the key issues of distributed multistatic radar is transmitting distinguishable waveforms and achieving a high signal-to-noise ratio (SNR) at the receivers. Deployed waveforms like the linear frequency modulated (LFM) waveform [7], orthogonal frequency division multiplexing (OFDM) waveforms [8] or a combination of them, though able to obtain an impulse-like output through matched filtering, need well-separated carrier frequencies for individual antennas to reduce cross-antenna interference in the matched filtering, necessitating a large frequency band for the centralized signal processing.

In this paper, an alternative waveform scheme built on mutually-orthogonal complementary sets is proposed for a distributed multistatic radar. Signal separation between antennas is established through complementary sets, which reduce the frequency band requirement as an identical carrier frequency is used in a centralized signal processing environment. We give a theoretical analysis of the influence of carrier frequencies and phases on range sidelobe suppression using complementary sets, validated by our simulations of multiple target illumination. This varies from Searle et al. [9,10], in that we avoid nonlinear processing of the complementary sets. Such processing can sometimes cause loss of target information [11].

## 2. Mutually Orthogonal Complementary Sets

Let $\left(A_{1},A_{2}\right)$ be a Golay complementary pair, in which $A_{1}$ and $A_{2}$ are both $L_{0}\times 1$ sequences with individual chip values equal to ±1 [12]. A mutually-orthogonal complementary set of sequences $\Delta^{\prime }$ of size $4\times 4\times 2L_{0}$ can then be constructed using the pair $\left(A_{1},A_{2}\right)$ as follows [13].

(1) $$\Delta^{\prime }=\left[\begin{array}{cc} \Delta \Delta & (-\Delta )\Delta \\ (-\Delta )\Delta & \Delta \Delta \end{array}\right],$$

where:

$$\Delta =\left[\begin{array}{cc} A_{1} & \tilde{A_{2}}\\ A_{2} & -\tilde{A_{1}} \end{array}\right]$$

is a $2\times 2\times L_{0}$ matrix and:

$$\Delta \Delta =\left[\begin{array}{cc} A_{1}A_{1} & \tilde{A_{2}}\tilde{A_{2}}\\ A_{2}A_{2} & \left(-\tilde{A_{1}}\right)\left(-\tilde{A_{1}}\right) \end{array}\right],$$

and we write $\tilde{A}$ for the sequence obtained by reversing the order of the elements of *A*, −A for the negation of *A* in elements and $A_{1}A_{2}$ for the concatenation of two sequences $A_{1}$ and $A_{2}$. Given a new Δ matrix equal to the generated $\Delta^{\prime }$, we are able to reuse (1) to generate higher dimension $\Delta^{\prime }$. By using (1) *r* times, we obtain a $\Delta^{\prime }$ matrix of size $M\left(=2^{r+1}\right)\times M\times L\left(=2^{r}L_{0}\right)$ given by:

(2) $$\Delta^{\prime }\left(:,:,l\right)=\left[\begin{array}{cccc} a_{11}\left(l\right) & a_{12}\left(l\right) & \ldots & a_{1M}\left(l\right)\\ a_{21}\left(l\right) & a_{22}\left(l\right) & \ldots & a_{2M}\left(l\right)\\ \vdots & \vdots & & \vdots \\ a_{M1}\left(l\right) & a_{M2}\left(l\right) & \ldots & a_{MM}\left(l\right) \end{array}\right].$$

where $a_{ij}$ is a 1×1×L binary sequence with values ±1, representing the row *i* column *j* entry of the matrix $\Delta^{\prime }$. The chip length of $a_{ij}$ (i.e., the length of $a_{ij}\left(l\right)$) is $T_{c}$, l=0,1,…,L−1. For example, a 4×4×4 $\Delta^{\prime }$ matrix can be generated from a Golay complementary pair $\left(A_{1}=++,A_{2}=+-\right)$ with $L_{0}=2$ by repeating (1) r=1 times:

$$\begin{array}{cc} \Delta^{\prime } & =\left[\begin{array}{cccc} a_{11} & a_{12} & a_{13} & a_{14}\\ a_{21} & a_{22} & a_{23} & a_{24}\\ a_{31} & a_{32} & a_{33} & a_{34}\\ a_{41} & a_{42} & a_{43} & a_{44} \end{array}\right]\\ & =\left[\begin{array}{cccc} ++++ & -+-+ & --++ & +--+\\ +-+- & ---- & -++- & ++--\\ --++ & +--+ & ++++ & -+-+\\ -++- & ++-- & +-+- & ---- \end{array}\right]. \end{array}$$

where “+” and “−” represent the chips of values ±1, respectively. $\Delta^{\prime }$ has the following properties. For arbitrary rows i,j (i≠j),

(3) $$\left\{\begin{array}{c} \sum_{p=1}^{M}C_{a_{ip},a_{ip}}\left(k\right)=ML\delta \left(k\right);\\ \sum_{p=1}^{M}C_{a_{ip},a_{jp}}\left(k\right)=0. \end{array}\right.$$

where:

$$C_{a_{ip},a_{jp}}\left(k\right)=\sum_{l=0}^{L-1}a_{ip}\left(l\right)a_{jp}^{*}\left(l-k\right)$$

represents the value of the cross-correlation of sequences $a_{ip}$ and $a_{jp}$ at index *k*, k=−(L−1), −(L−2),…,L−1. The superscript “*” denotes complex conjugation, and δ(k) is the Kronecker delta function. These properties are also satisfied for arbitrary columns.

## 3. Radar System Model

**Figure 1:**
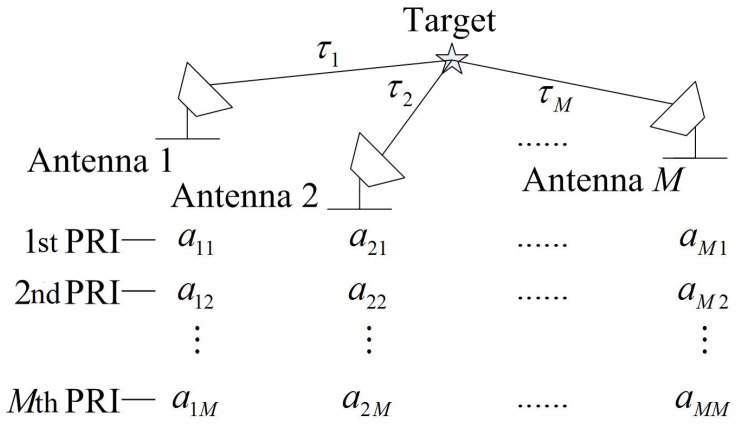
The schematic figure of the radar detection scenario.

The $\Delta^{\prime }$ matrix is used to construct the transmitted waveforms for a distributed multistatic radar system with antennas 1,2,…,m,…,M that can both transmit and receive signals. Consider a static point target illuminated by the radar system as shown in Figure 1, where $\tau_{1}$ to $\tau_{M}$ are the round-trip delay values between the target and each antenna. Antenna *m* transmits the sequence $a_{mp}$ in the *p*-th (p=1,2,…,M) pulse repetition interval (PRI) and receives echoes from all antennas, which are the radar returns’ delayed versions of $\left[a_{1p},\cdots ,a_{Mp}\right]$. The complementary sets on each antenna are modulated by a baseband pulse Ω(t), yielding the following time domain waveforms:

(4) $$a_{mp}\left(t\right)=\sum_{l=0}^{L-1}a_{mp}\left(l\right)\Omega \left(t-lT_{c}\right),$$

where $\int_{-T_{c}/2}^{T_{c}/2}|\Omega \left(t\right){|}^{2}dt=1$. Ideally, Ω(t) is a rectangular pulse, and this is used in our simulations for simplicity, but in a real system, the rectangular pulse would typically be replaced by another pulse shape, such as a raised cosine or Gaussian pulse to reduce the bandwidth requirement.

$a_{mp}\left(t\right)$ is modulated by the carrier frequency $f_{c_{m}}$ and a phase $\phi_{m}$ at antenna *m* in the transmitted waveforms $a_{mp}\left(t\right)e^{j2\pi f_{c_{m}}\left(t+\phi_{m}\right)}$. The signal received by antenna *m* in the *p*-th PRI is:

(5) $$y_{mp}\left(t\right)=\sum_{i=1}^{M}a_{ip}\left(t-\frac{\tau_{i}+\tau_{m}}{2}\right)e^{j2\pi f_{c_{i}}\left(t-\frac{\tau_{i}+\tau_{m}}{2}+\phi_{i}\right)},$$

and the output of antenna *m* for the *p*-th PRI after demodulation with $e^{-j2\pi f_{c_{m}}\left(t+\phi_{m}\right)}$ and match filtering with $a_{mp}\left(t\right)$ is:

(6) $$z_{mp}\left(t\right)=\sum_{k=-L+1}^{L-1}y_{mp}\left(t\right)e^{-j2\pi f_{c_{m}}\left(t+\phi_{m}\right)}a_{mp}^{*}\left(t\right),$$

where the superscript “*” denotes complex conjugation. Summing the results over all *M* PRIs, we obtain a final output of antenna *m*:

(7) $$\begin{array}{cc} z_{m}\left(t\right) & =\sum_{k=-L+1}^{L-1}\sum_{p=1}^{M}y_{mp}\left(t\right)e^{-j2\pi f_{c_{m}}\left(t+\phi_{m}\right)}a_{mp}^{*}\left(t\right)\\ & =\sum_{k=-L+1}^{L-1}\sum_{p=1}^{M}\sum_{i=1}^{M}a_{ip}\left(t-\frac{\tau_{i}+\tau_{m}}{2}\right)a_{mp}^{*}\left(t\right)e^{j2\pi f_{c_{i}}\left(t-\frac{\tau_{i}+\tau_{m}}{2}+\phi_{i}\right)}e^{-j2\pi f_{c_{m}}\left(t+\phi_{m}\right)}. \end{array}$$

## 4. Influence of Carrier Frequencies and Phases

As mentioned before, $f_{c_{m}}$ is often varied across antennas for traditional waveforms like the LFM waveform in order to guarantee orthogonality. This requires a wide frequency band for transmission of the waveforms. Additionally, the different carrier-dependent phases $\phi_{m}$ may also complicate the post-processing of radar returns (e.g., may decrease the output of coherent integration). However, the transmission of complementary sets with identical carrier frequency for all antennas, i.e., $f_{c_{m}}=f_{c}$, results in (7) becoming:

(8) $$z_{m}\left(t\right)=\sum_{i=1}^{M}\sum_{k=-L+1}^{L-1}\left\{\begin{array}{c} \sum_{p=1}^{M}C_{a_{mp},a_{ip}}\left(k\right)C_{\Omega }\left(t-\frac{\tau_{i}+\tau_{m}}{2}-kT_{c}\right)\\ e^{-j2\pi f_{c}\left(\frac{\tau_{i}+\tau_{m}}{2}+\phi_{m}-\phi_{i}\right)} \end{array}\right\},$$

where $C_{\Omega }$ denotes the autocorrelation of the baseband pulse and:

(9) $$\sum_{p=1}^{M}C_{a_{mp},a_{ip}}\left(k\right)=\left\{\begin{array}{cc} ML\delta (k) & i=m,\\ 0 & i\neq m. \end{array}\right.$$

As a consequence, the complementary sets achieve an impulse output. In addition, at least in theory, the phase shifts $\phi_{m}-\phi_{i}$ do not influence the level of range sidelobes, nor the further signal processing.

Based on previous discussions, the following remarks can be made:

(a) Theoretically, complementary sets are free of cross-antenna interference, as well as range sidelobes (induced by the cross terms of cross-correlation) when an identical carrier frequency is used for all antennas, and the results are not influenced by the phase differences between antenna carriers. In this case, the radar has an equivalent pulse width of $2T_{c}$.

(b) Compared to conventional LFM or OFDM waveforms, this alternative scheme reduces the frequency band requirement for transmission in the centralized radar signal processing system at the cost of increased radar illumination time (needs more accumulation of pulses) for the same pulse width and sampling rate.

(c) Remark (a) will not hold for complementary sets if the carrier frequency variation across antennas is large.

## 5. Simulation and Discussion

In the simulation, we consider a scenario with four antennas. A 4×4×L $\Delta^{\prime }$ matrix of complementary sets is generated for transmission. The bandwidth of the radar is B=5MHz. The sampling rate is $f_{s}=2B$. The PRI is T=5μs. Each entry of the matrix $\Delta^{\prime }$ has L=64 chips of values ±1 in the chip interval $T_{c}=0.1\;\mu s$. The locations of Antennas 1—4 in the scenario are set to be (0,0)m, (1500,300)m, (2000,1000)m, (1400,800)m, respectively. Two point stationary targets are assumed at (700,600)m and (1000,400)m with the magnitude 0dB and −20dB, respectively. Radar returns at all antennas are contaminated by complex Gaussian zero-mean white noise E∼CN(0,1) with a mean magnitude of −10dB.

We study three cases for different carrier frequency and phase configurations:

$$\begin{array}{cc} \left(1\right)f_{c_{1}} & =f_{c_{2}}=f_{c_{3}}=f_{c_{4}}=1\;\mathrm{GHz};\phi_{1}=\phi_{2}=\phi_{3}=\phi_{4}=0.\\ \left(2\right)f_{c_{1}} & =1\;\mathrm{GHz},f_{c_{2}}=1\;\mathrm{GHz}+2\;\mathrm{MHz},f_{c_{3}}=1\;\mathrm{GHz}+4\;\mathrm{MHz},f_{c_{4}}=1\;\mathrm{GHz}+6\;\mathrm{MHz};\\ \phi_{1} & =\phi_{2}=\phi_{3}=\phi_{4}=0.\\ \left(3\right)f_{c_{1}} & =1\;\mathrm{GHz}+\eta_{1},f_{c_{2}}=1\;\mathrm{GHz}+\eta_{2},f_{c_{3}}=1\;\mathrm{GHz}+\eta_{3},f_{c_{4}}=1\;\mathrm{GHz}+\eta_{4};\\ \phi_{1} & =0,\phi_{2}=\left(\pi /5\right)\;\mathrm{rad},\phi_{3}=\left(\pi /3\right)\;\mathrm{rad},\phi_{4}=\left(\pi /2\right)\;\mathrm{rad}. \end{array}$$

The random parameters $\eta_{1}$, $\eta_{2}$, $\eta_{3}$ and $\eta_{4}$, which reflect the possible frequency jitter or drift invoked by the radar system or environment, are in the frequency interval [−10kHz,10kHz] [14].

**Figure 2:**
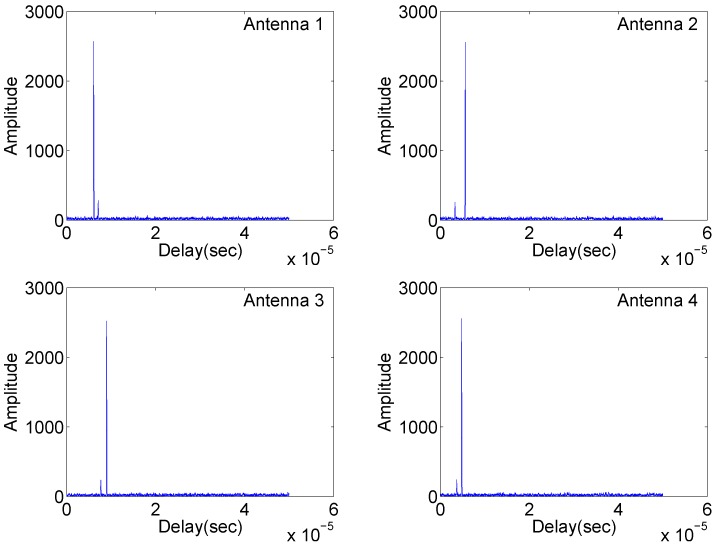
Matched filtering outputs of the four antennas in Case (1).

**Figure 3:**
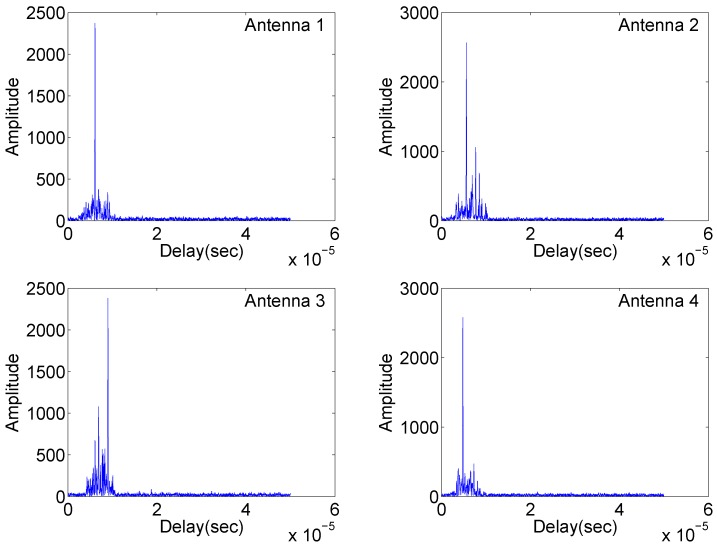
Matched filtering outputs of the four antennas in Case (2).

**Figure 4:**
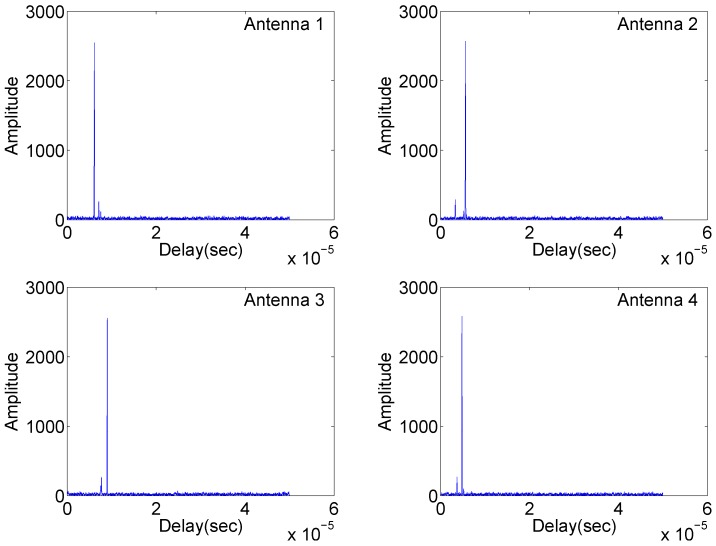
Matched filtering outputs of the four antennas in Case (3).

Simulation results are shown in Figure 2, Figure 3 and Figure 4.

Figure 2 illustrates the matched filter outputs at the four antennas with identical carrier frequencies, where radar returns are “free” of range sidelobes and cross-antenna interference due to the complementary set properties mentioned in the first remark. Two targets are clearly separated. However, in Figure 3, significant range sidelobes induced by the carrier frequency variation are observed. Such sidelobes can submerge weak targets and make it difficult to detect them. The results in Figure 4 demonstrate resilience to jitter or drift of carrier frequency and phase differences at the matched filter outputs. The overall simulation results suggest that waveforms based on complementary sets are practical alternatives for distributed multistatic radar systems, producing strong performance while retaining identical carrier frequencies across all antennas.

## 6. Conclusions

We have studied an alternative waveform scenario to traditional schemes composed of mutually-orthogonal complementary sets for distributed multistatic radar illumination. Our simulation shows that under identical carrier frequency across antennas, phase differences and/or small frequency drift or jitter cause little effect on the output SNR for all antennas, while increased range sidelobes arise if antennas are working at significantly different carrier frequencies.
